# The Prevalence and Associated Factors of Self-Reported Knee Injuries Among Amateur Players in Saudi Arabia

**DOI:** 10.3390/healthcare14172738

**Published:** 2026-08-27

**Authors:** Ibrahim Eissa J. Alfaifi, Msaad Alzhrani

**Affiliations:** 1 General Administration of Medical Services, Tabuk University, Tabuk 47512, Saudi Arabia; ialfaifi@ut.edu.sa; 2 Department of Physical Therapy and Health Rehabilitation, College of Applied Medical Sciences, Majmaah University, Al Majmaah 11952, Saudi Arabia

**Keywords:** knee injury, prevalence, associated factors, amateur players, football, Saudi Arabia, KOOS

## Abstract

**Highlights:**

**What are the main findings?**

- In this convenience sample, 56.8% of amateur players reported a lifetime history of knee injury (155/273; 95% CI 50.8–62.5%).
- Football was the only sport consistently associated with higher odds of self-reported lifetime knee injury in both bivariate analysis (OR = 2.42, 95% CI 1.38–4.22; *p* = 0.002) and multivariable Firth analysis (aOR = 2.60, 95% CI 1.35–5.13; *p* = 0.004).

**What are the implications of the main findings?**

- Together with external injury prevention evidence, these findings may help inform knee injury prevention, progressive conditioning, and early rehabilitation initiatives in amateur football settings.
- Clear data cleaning procedures are important when self-administered injury history questionnaires are used.

**Abstract:**

**Background/Objectives:** Saudi evidence on self-reported knee injuries among amateur sport participants is limited. We estimated lifetime knee injury prevalence and associated factors. **Methods:** This cross-sectional study used non-probability convenience sampling to recruit amateur players aged 18–60 years without chronic disease. Data were collected in 2022 using an Arabic study questionnaire and the Saudi Arabic Knee Injury and Osteoarthritis Outcome Score. The primary outcome was a broadly defined, self-reported lifetime knee injury history. Associations were estimated using logistic and Firth penalized logistic regression. **Results:** Of 462 submissions after the eligibility gateway, 273 were eligible and internally consistent. Most participants were male (89.4%), and football was the most frequently reported sport (74.7%). Overall, 155 participants reported a lifetime knee injury (56.8%; 95% CI 50.8–62.5%). In the adjusted model (n = 269), football participation (aOR = 2.60, 95% CI 1.35–5.13; *p* = 0.004) and older age (aOR per year = 1.04, 95% CI 1.01–1.08; *p* = 0.023) were associated with higher odds. Estimates for male sex and high exercise intensity were imprecise. **Conclusions:** More than half of this convenience sample reported a lifetime knee injury. Football was the most consistent correlate. These cross-sectional findings are neither causal nor nationally representative.

## 1. Introduction

Sporting activities have become an important component of modern life, with increasing participation in sports clubs, fitness studios, and recreational exercise. Although sports participation has recognized health benefits, it also exposes participants to contexts in which injuries can occur. Contemporary sports injury surveillance defines an injury broadly as tissue damage or another disturbance of normal physical function attributable to participation in sport, irrespective of whether medical attention or time loss occurs [1].

Reported sports injury occurrence varies across settings and depends on the study design and epidemiological measure used. In a prospective cohort in Western Australia, incidence ranged from 12.1 to 20.3 injuries per 1000 participation hours across four sports, whereas 28.2% of girls and 35.9% of boys in a Swiss school survey reported at least one sports injury during the preceding year [2,3]. In Saudi Arabia, national participation data indicate substantial engagement in sport and physical activity [4], while the local sports injury literature indicates that participation in recreational exercise settings is accompanied by exposure to sports-related injuries [5]. Football is widely practiced in Saudi Arabia, and Saudi studies have documented injury patterns among football participants, including knee injuries [6,7].

The knee joint is particularly vulnerable in sports that involve running, jumping, rapid deceleration, pivoting, and physical contact. The lower extremity is commonly affected in such sports because of high mechanical loading and frequent changes in direction [8]. Knee injuries represent an important component of the sports injury burden among adolescents, although epidemiological estimates vary widely by sport and study design [9].

The knee is stabilized by the articular geometry, menisci, and major ligamentous structures, including the anterior cruciate ligament, posterior cruciate ligament, medial collateral ligament, and lateral collateral ligament. Injuries to these structures may cause pain, instability, and reduced function [10,11]. Intrinsic and extrinsic factors may influence injury occurrence, including age, sex, previous injury, biomechanics, sports environment, equipment, and rules [12]. Previous knee injury and poorer knee function have also been reported as risk factors for subsequent knee injuries in youth team sport athletes [13].

Previous Saudi studies have reported knee injuries among male college students in Riyadh and university students in Jazan [14,15]. However, evidence remains limited for broader community amateur or recreational sport participants recruited from clubs, public playgrounds, and online channels. Such participants may differ from university-based samples in age distribution, sport mix, training exposure, supervision, and access to rehabilitation. Because cross-sectional data cannot establish temporal causality, the current study focuses on the sample-specific prevalence of self-reported lifetime knee injury history and on associated factors rather than causal risk factors. This study aimed to assess the prevalence of self-reported lifetime knee injury history and associated factors among amateur players in Saudi Arabia. No formal directional hypothesis was prespecified.

## 2. Materials and Methods

### 2.1. Study Design and Participants

This cross-sectional study was conducted among amateur players from different sports in Saudi Arabia between 28 July 2022 and 10 November 2022. Data collection began after ethical approval was obtained on 28 July 2022 and continued until 10 November 2022. Participants were recruited through non-probability convenience sampling from sports clubs, public playgrounds, and online channels using Google Forms Google Forms (Google LLC, Mountain View, CA, USA; https://workspace.google.com/products/forms/; accessed on 17 August 2026). Exact counts of recruitment sites, public playgrounds, and online channels were not recorded. A conventional response rate could not be calculated because the number of individuals who received or viewed the open survey invitation was not recorded.

The target population comprised adults aged 18–60 years participating in amateur sport in Saudi Arabia. Eligibility required age 18–60 years, amateur player status, current participation in sport or physical activity, absence of chronic disease, and willingness to provide informed consent. Before questionnaire access, prospective participants were required to affirm all five criteria through a mandatory eligibility gateway. Only individuals who affirmed all criteria could access the 26-item questionnaire. The platform did not retain the number of individuals who did not proceed or the specific criterion they did not meet.

The online form then displayed the Arabic participant information text reproduced verbatim in Supplementary Material S1. Age, amateur status, current sport or physical activity participation, and formal informed consent were recorded again as verification items; formal consent was item 1. A “No” response did not technically terminate the form but was treated as refusal of consent. Because the form remained open, fields entered after item 1 initially appeared in the raw export. All questionnaire information from the nine non-consented submissions was subsequently deleted, consistent with the approved ethics protocol. No individual-level or substantive information from these submissions was included in descriptive, inferential, sensitivity, or supplementary analyses. Only the aggregate number of consent refusals (n = 9) was retained for participant flow and exclusion reporting.

An amateur player was operationally defined as an individual participating recreationally or through a non-professional local club or public playground, without professional or advanced competitive status. The study is reported in accordance with the Strengthening the Reporting of Observational Studies in Epidemiology (STROBE) recommendations for cross-sectional studies, and the completed checklist is supplied separately.

### 2.2. Sample Size and Data Cleaning

The original sample size calculation used the available national estimate of 5.91 million Saudi citizens participating in sports activities as a pragmatic large-population input derived by the authors from national statistics [4]. Using the Raosoft Sample Size Calculator (Raosoft, Inc., Seattle, WA, USA; original website: http://www.raosoft.com/samplesize.html, accessed on 11 July 2021; archived version: https://web.archive.org/web/20240217233316/http://www.raosoft.com/samplesize.html, accessed on 17 August 2026) [16], a 95% confidence level, 5% margin of error, and prespecified response distribution of 70% produced a recruitment target of 323 participants. The 70% value was an a priori planning assumption rather than an established knee injury prevalence estimate. Because recruitment used non-probability convenience sampling, this calculation served only as a pragmatic recruitment target and does not imply that the final sample achieved a population-level 5% margin of error. The final analytic sample of 273 was 50 participants (15.5%) below this target.

Among 462 submitted records, 171 were excluded hierarchically during eligibility verification: 9 because formal consent was refused or not provided; 129 for amateur-status reasons, comprising 66 professional players and 63 non-practicing individuals; 20 after sport participation pattern verification, comprising 11 reporting professional athlete status and 9 reporting no sport participation; and 13 because age was outside 18–60 years or the age entry was invalid. All questionnaire information from the nine non-consented submissions was deleted, consistent with the approved ethics protocol, and only the aggregate refusal count was retained. This left 291 eligible records.

Data consistency screening then excluded 18 records because the primary lifetime knee injury outcome could not be classified reliably. All 18 participants answered “No” to the question “Have you ever had a knee injury?” but supplied contradictory injury-related information. Eight reported that an injury had resulted from a clash with another player, and ten provided other substantive information, including a named injury type, healthcare or physiotherapy, persistent pain, recovery details, or treatment information. The final primary analytic sample therefore comprised 273 participants. The complete eligibility and data cleaning audit is provided in Supplementary Material S2; the extreme-case classification analyses are reported in Section 3.2.

**Figure 1:**
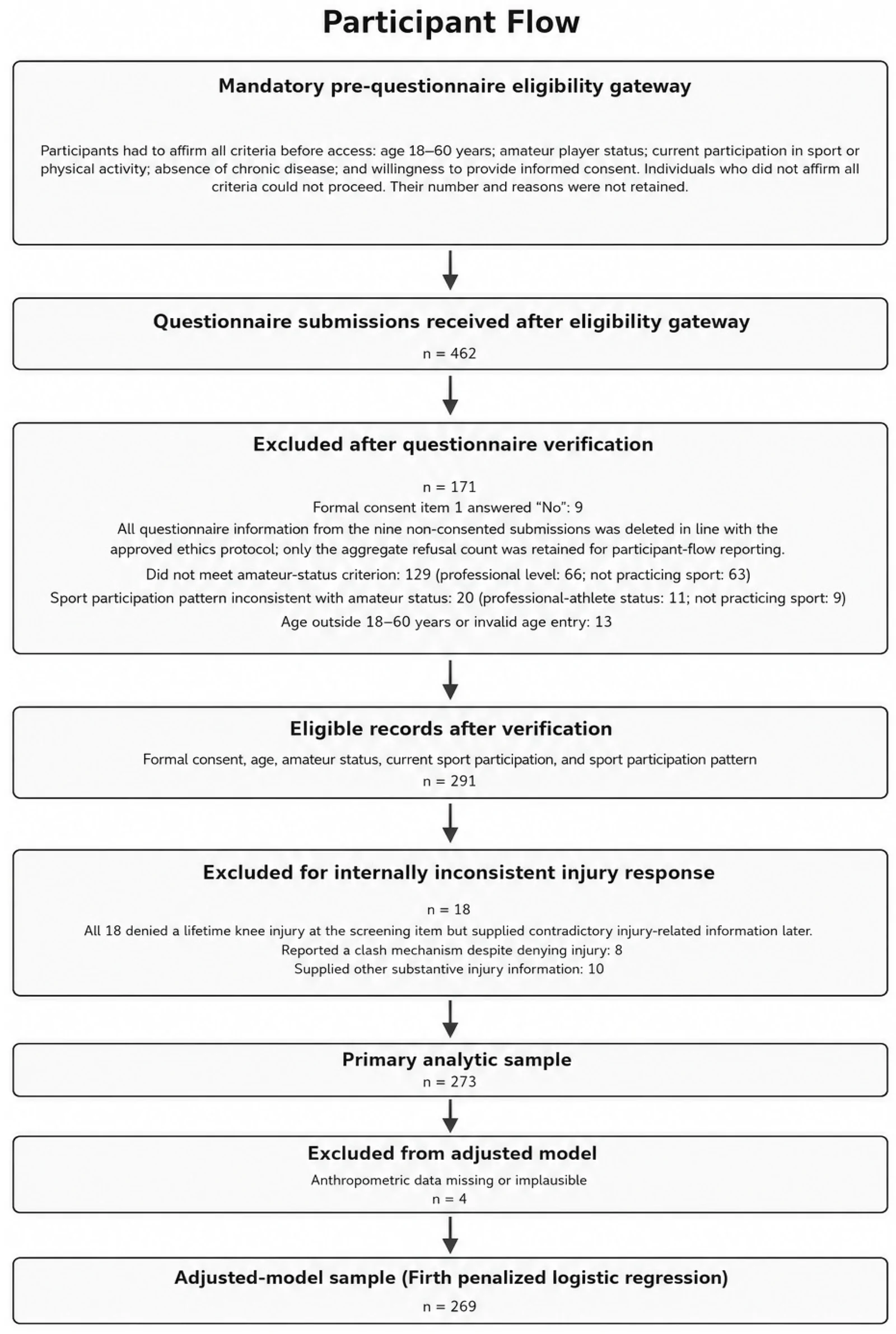
Participant flow diagram. The platform did not retain the number of individuals who did not affirm the mandatory pre-questionnaire eligibility statement. Subsequent exclusions were applied hierarchically; each submitted record was counted once under the first applicable criterion. BMI, body mass index.

The questionnaire was not reopened solely to replace records excluded after data review. Four participants were excluded only from analyses requiring BMI because anthropometric data were unusable: two contained non-numeric height entries, one recorded a height of 70 cm, and one recorded a weight of 780 kg. Two heights entered in meters, “1.84” and “1.73 m,” were converted to 184 cm and 173 cm and retained. The adjusted regression sample therefore included 269 participants, comprising 154 with and 115 without a reported lifetime knee injury. Participant flow is presented in Figure 1.

### 2.3. Questionnaire

#### 2.3.1. Researcher-Developed Study-Specific Questionnaire

The data collection instrument included a 26-item researcher-developed, literature-informed Arabic questionnaire organized into two sections. The first section contained 11 items on consent, participant characteristics, region, sporting level, sports practiced, and training frequency, duration, and intensity. The second section contained 15 items on health status, sport participation pattern, lifetime knee injury history, injury mechanism and type, healthcare use, treatment, pain, recovery, injury prevention, and knee range of motion. Development of these study-specific domains was informed by the relevant Saudi sports injury literature, including Alaqil et al. (2021), Almaawi et al. (2020), and Moafa et al. (2022) [5,14,15].

The original questionnaire explicitly defined a knee injury as any injury affecting the knee, ranging from a contusion or bruise to the most severe injuries. The primary outcome therefore represented a broad self-reported lifetime history of knee injury. Reported injuries were not clinically verified by the research team, the sport at the time of injury was not recorded, and the questionnaire did not require participants to anchor follow-up injury details to the most recent or most severe injury.

The questionnaire was pilot tested among 25 eligible amateur players to assess item clarity, comprehensibility, and completion feasibility. No items were modified after the pilot; the instrument was therefore identical for all participants, and pilot responses were retained in the main analysis. The study-specific items were developed and administered directly in Arabic; therefore, translation and back-translation were not applicable. The study-specific questionnaire was not formally assessed through expert-panel content validation, test–retest reliability, or psychometric testing. Consequently, its content validity, reproducibility, and measurement performance were not formally established, which represents an important methodological limitation. The complete researcher-developed questionnaire is provided in Supplementary Material S1.

#### 2.3.2. Saudi Arabic KOOS

Participants additionally completed the Saudi Arabic Knee Injury and Osteoarthritis Outcome Score (KOOS), which has undergone cross-cultural adaptation and validation in Saudi Arabic [17]. KOOS items refer to the previous week and are temporally distinct from the primary lifetime injury history outcome. The Symptoms, Activities of Daily Living, Sport and Recreation, and Quality of Life subscales were administered in full. During transfer of the paper questionnaire to the online form, two Pain items (P3 and P7) were inadvertently omitted. This was an administration construction error rather than an intentional modification of KOOS. The resulting score was calculated from the seven administered Pain items and reported separately as the seven-item Pain score. It is not equivalent to, and should not be interpreted as, the validated nine-item KOOS Pain subscale. Internal consistency of the seven items within this sample was Cronbach alpha = 0.871, with corrected item-total correlations of 0.60–0.71; however, these findings do not establish equivalence to or validation of the standard Pain subscale. The four fully administered KOOS subscales and the seven-item Pain score met the minimum available-item requirement used for scoring, and no participant-level score was excluded because of insufficient item responses [18].

At the time of data collection in 2022, KOOS was publicly available for use through the KOOS User’s Guide and website [18]. Mapi Research Trust currently manages KOOS distribution and licensing and describes itself as the official licensor and distributor of COAs on behalf of developers and copyright holders [19,20]. Use in the present work is covered by User License Agreement No. 142688. KOOS© Ewa Maria Roos, 1998. KOOS items are not reproduced in the Supplementary Material under the current agreement.

### 2.4. Statistical Analysis

All statistical analyses were performed in R version 4.6.1 (R Foundation for Statistical Computing, Vienna, Austria) [21]. The readxl package version 1.5.0 was used for data import [22], and logistf version 1.26.1 was used for Firth penalized logistic regression, profile penalized-likelihood confidence intervals, and penalized likelihood-ratio tests [23]. All other procedures used the base R stats package or purpose-written code.

Wilson score confidence intervals were calculated using the closed-form score expression and the standard normal quantile function. Crude odds ratios and Wald 95% confidence intervals were obtained from univariable logistic regression models fitted using glm, with likelihood-ratio *p*-values obtained using anova. Complete and quasi-complete separation were assessed by inspecting minimum cell counts for all binary terms. Variance inflation factors were derived from auxiliary linear regressions of each predictor on the remaining predictors. Linearity in the logit was assessed by adding an x·log(x) term to the logistic model and testing its coefficient. The overall adjusted-model test used logistftest, with the design matrix and Jeffreys penalty held fixed.

Bootstrap resampling used sample with 1000 resamples and random seed 20260724, with the penalized model refitted in each replicate. KOOS response options were matched after removal of Arabic diacritics and unification of alif variants so that spelling variants of the same response category were coded identically; subscale scores were then computed according to the KOOS User’s Guide, Version 1.1 scoring definitions [18]. Cronbach alpha was calculated from item and total-score variances, and corrected item-total correlations were calculated as the correlation between each item and the sum of the remaining items. Mann–Whitney U tests were performed using wilcox.test, and effect size r = |Z|/√N was derived from the rank-sum statistic with correction for ties rather than back-calculated from the *p*-value.

Categorical variables are presented as frequencies and percentages. The primary outcome was self-reported lifetime history of knee injury under the broad participant-facing definition described above. Sport and training variables were phrased in the present tense and described participants’ current or usual participation at survey completion, without a defined retrospective exposure period; therefore, temporal ordering relative to a previous injury could not be established. Participants could select more than one sport, so sport indicators were multi-response and not mutually exclusive. Checkbox selections and free-text sport responses were harmonized using a conservative coding dictionary: direct lexical equivalents and recognizable spelling variants of a listed sport were assigned to that category, whereas distinct exercise modalities were retained as other activities. For strengthening exercises, direct equivalents included weightlifting, weights, resistance training, iron training, and bodybuilding; CrossFit and calisthenics were retained as other activities. The complete coding dictionary, including the exact free-text matching terms and category assignments, is provided in Supplementary Material S2. The primary lifetime knee injury proportion was reported with a Wilson 95% confidence interval. Two extreme-case sensitivity analyses were conducted for the 18 internally inconsistent records: all were treated as not injured in one analysis and as injured in the other. For predictors with more than two categories, an overall likelihood-ratio *p*-value was reported for the variable as a whole; for binary predictors, a single likelihood-ratio *p*-value was reported for the corresponding contrast. Sports were analyzed inferentially only when both outcome cells contained at least 10 participants; sports with fewer than 10 participants in either outcome cell were summarized descriptively without odds ratios or inferential *p*-values. No subgroup or interaction analyses were prespecified or performed. Because recruitment used non-probability convenience sampling, no sampling weights or design-based variance corrections were applied.

The primary adjusted model used Firth penalized logistic regression to reduce small-sample bias and improve estimation in the presence of sparse categories [24,25]. The predictor set was specified a priori in the study proposal based on the previous literature and clinical relevance and represented demographic, anthropometric, training-related, and sport participation characteristics potentially associated with self-reported lifetime knee injury history. It included age, sex, BMI, weekly exercise frequency, exercise duration, exercise intensity, sport participation pattern, football, running, strengthening exercises, volleyball, and cycling. For descriptive and crude analyses, age was grouped into 18–24, 25–34, 35–44, and 45+ years to provide interpretable strata while maintaining usable category sizes; BMI was grouped into the categories shown in Table 1. Age and BMI were retained as continuous variables in the adjusted model to avoid information loss from categorization. Except for four participants with unusable anthropometric data, all predictors in the adjusted model were complete. These four participants were excluded using complete-case analysis, and no values were imputed. The adjusted model therefore included 269 participants. Odds ratios are reported with profile penalized-likelihood 95% confidence intervals, and *p*-values are penalized likelihood-ratio tests.

The model contained 15 estimated predictor parameters and 115 observations in the smaller outcome group, corresponding to 7.7 smaller-outcome observations per parameter. The predictor set and full Firth penalized model were prespecified in the study proposal. The maximum-likelihood–Firth comparison, reduced Firth model, bootstrap stability analysis, and KOOS between-group comparison were added during revision and were interpreted as sensitivity, exploratory, or post hoc analyses rather than confirmatory analyses. The maximum-likelihood versus Firth comparison is provided in Supplementary Material S3, and the reduced Firth model and 1000-resample bootstrap analysis are provided in Supplementary Material S4. Sparse categories, including age 45+, daily exercise, high exercise intensity, the female reference group for sex, and exercise duration > 2 h, were interpreted cautiously.

KOOS values were calculated according to the KOOS User’s Guide, Version 1.1 and transformed to a 0–100 scale, with 100 indicating no knee problems [18]. The seven-item Pain score was calculated from the seven administered Pain items and was not treated as equivalent to the complete nine-item KOOS Pain subscale. The 50% available-item requirement was applied to the four fully administered subscales and the seven-item Pain score; no participant-level score was excluded because of insufficient responses. Where a score was based on a partial but sufficient participant response set, it was calculated as the mean of the available items in accordance with the scoring guidance. For one Sport and Recreation item concerning twisting or pivoting on the affected knee, “Not applicable” responses were treated as unavailable item responses; all affected participants completed the remaining four items and met the scoring requirement. During revision, a post hoc secondary analysis compared KOOS values between participants with and without self-reported lifetime knee injury history. This analysis examined whether lifetime injury history was associated with current knee symptoms and function and was not intended to establish diagnostic validity or temporal correspondence. Statistical significance was set at *p* < 0.05.

Potential sources of bias were addressed through a participant-facing knee injury definition, pre-questionnaire eligibility screening, hierarchical eligibility and response consistency rules, extreme-case classification bounds for inconsistent records, Firth penalization for sparse adjusted estimates, and cautious interpretation of temporally mismatched and overlapping sport exposures. These procedures could reduce some forms of misclassification and small-sample bias but could not eliminate selection bias, recall bias, or exposure misclassification.

## 3. Results

### 3.1. Participant Flow and Sample Characteristics

Of 462 submitted records, 171 were excluded during eligibility verification and 18 because they denied a lifetime knee injury but supplied contradictory injury-related information, preventing reliable classification of the primary outcome. The final primary analytic sample comprised 273 participants. Four participants with unusable anthropometric data were excluded only from analyses requiring BMI, resulting in an adjusted-model sample of 269, including 154 participants with and 115 without a reported lifetime knee injury.

The sample was predominantly male (244/273, 89.4%) and included a high proportion of football participants (204/273, 74.7%), features that are important when interpreting the subsequent analyses. The largest age group was 25–34 years (137/273, 50.2%), and the Western region was the most represented region (106/273, 38.8%). Table 1 and Table 2 summarize participant and sport characteristics.

Football was the most frequently reported sport, followed by running (90/273, 33.0%), strengthening exercises (86/273, 31.5%), and volleyball (69/273, 25.3%). Participants could report more than one sport; therefore, sport indicators were overlapping and not mutually exclusive. Most participants exercised 1–3 days per week (170/273, 62.3%), exercised for 1–2 h per session (141/273, 51.6%), and reported medium exercise intensity (176/273, 64.5%).

### 3.2. Lifetime Knee Injury Proportion and Clinical Characteristics

Among the 273 included participants, 155 reported a lifetime knee injury, corresponding to 56.8% (95% CI 50.8–62.5%). If all 18 internally inconsistent records were classified as not injured, the estimate was 53.3% (155/291; 95% CI 47.5–58.9%); if all were classified as injured, it was 59.5% (173/291; 95% CI 53.7–64.9%). The estimates therefore varied by 6.2 percentage points, and the primary estimate remained within these bounds. However, these extreme-case estimates address only uncertainty arising from classification of the 18 inconsistent records. They do not resolve uncertainty related to convenience sampling, recall, the broad injury definition, or the absence of clinical verification.

Among participants reporting an injury, 80 (51.6%) stated that it resulted from a clash with another player during sports activity. The most frequently reported injury types were bruises (62/155, 40.0%), meniscus/cartilage injuries (50/155, 32.3%), and anterior cruciate ligament/anterior ligament injuries (45/155, 29.0%). Injury type was a multi-response item.

Medical care was reported by 115 injured participants (74.2%), physiotherapy by 77 (49.7%), and surgical care by 41 (26.5%). The mean pain score was 6.5 (SD 2.5) out of 10, the median was 7/10, 63 participants (40.6%) reported pain scores of 8–10, and 76 (49.0%) reported recovery lasting more than four weeks. All injury characteristics, healthcare use, pain, and recovery outcomes were self-reported and were not independently verified from clinical records. Table 3 summarizes these findings.

### 3.3. Crude Associations with Participant and Training Characteristics

In univariable analyses, male participants had higher crude odds of reporting a lifetime knee injury than female participants (cOR = 3.31, 95% CI 1.45–7.57; global likelihood-ratio *p* = 0.003). The global tests for age group, BMI category, exercise frequency, exercise duration, exercise intensity, and sport participation pattern did not provide strong evidence of overall differences between categories. Although injury proportions generally increased across age groups, the global age-group test was not statistically significant (*p* = 0.170). The complete crude estimates are presented in Table 4.

### 3.4. Crude Associations with Sport Participation

Football participation was associated with higher crude odds of reporting a lifetime knee injury (cOR = 2.42, 95% CI 1.38–4.22; *p* = 0.002). Using the harmonized sport indicators, strengthening exercises were not clearly associated with the outcome in the crude model (cOR = 1.54, 95% CI 0.91–2.61; *p* = 0.103). Running, volleyball, and cycling were also not clearly associated in univariable models. Sports with fewer than 10 participants in either outcome cell were summarized descriptively without odds ratios or inferential *p*-values (Table 5 and Table 6).

### 3.5. Adjusted Firth Penalized Logistic Regression

The full adjusted model included the predictors specified in the original study proposal. The model contained 15 estimated predictor parameters, with 115 observations in the smaller outcome group, corresponding to 7.7 smaller-outcome observations per parameter. Firth penalized logistic regression was used to reduce small-sample bias and improve stability in the presence of sparse categories. Using the harmonized sport indicators, the overall penalized likelihood-ratio test was statistically significant (χ^2^ = 36.29, df = 15; *p* = 0.002).

Football participation was associated with higher adjusted odds of self-reported lifetime knee injury (aOR = 2.60, 95% profile penalized-likelihood CI 1.35–5.13; *p* = 0.004). Each additional year of age corresponded to approximately 4.3% higher odds (aOR = 1.04, 95% CI 1.01–1.08; *p* = 0.023).

Male sex (aOR = 2.49, 95% CI 1.00–6.47; *p* = 0.049) and high rather than low exercise intensity (aOR = 3.53, 95% CI 1.03–13.58; *p* = 0.045) produced estimates above the null, but both were imprecise and had wide confidence intervals. The volleyball estimate was below the null (aOR = 0.54, 95% CI 0.29–1.00; *p* = 0.049), while cycling produced an estimate above the null with a confidence interval crossing 1 (aOR = 2.05, 95% CI 0.92–4.78; *p* = 0.080). Because these estimates were imprecise, several categories were sparse, and no multiplicity adjustment was applied, the findings for sex, high intensity, volleyball, and cycling are considered exploratory. BMI, exercise frequency, exercise duration, sport participation pattern, running, and strengthening exercises were not clearly associated with the outcome in the full penalized model.

The exploratory inverse volleyball coefficient should not be interpreted as a protective association. Sixty-one of the 69 volleyball participants (88.4%) also reported football, and the crude volleyball estimate was compatible with no association. The adjusted result may therefore reflect conditioning on overlapping sport indicators, residual confounding, or sampling variability (Table 7).

### 3.6. Model Diagnostics and Stability

No complete or quasi-complete separation was identified. The maximum variance inflation factor was 1.74, indicating no evidence of problematic conventional multicollinearity. The linearity-in-the-logit assessments were acceptable for age (Box–Tidwell *p* = 0.673) and BMI (*p* = 0.411).

The maximum-likelihood–Firth comparison showed modest shrinkage toward the null without reversal of coefficient direction. As a stability check rather than confirmatory evidence, the football odds ratio exceeded 1 in 999 of 1000 bootstrap resamples, with a median OR of 2.84 and a 2.5th–97.5th percentile interval of 1.43–5.98. The high-intensity estimate was directionally stable but substantially less precise.

The reduced Firth model was added during revision as an exploratory analysis and was not prespecified. It included age, sex, BMI, high exercise intensity, and football participation and was statistically significant overall (penalized likelihood-ratio χ^2^ = 24.19, df = 5; *p* < 0.001). Football remained associated with higher odds (aOR = 2.20, 95% CI 1.20–4.11; *p* = 0.011). The reduced model compared high intensity with low and medium intensity combined, whereas the full model compared high with low intensity; the two estimates are therefore not directly comparable. Complete results are provided in Supplementary Materials S3 and S4.

### 3.7. KOOS Secondary Comparison

In a post hoc analysis added during revision, participants reporting a lifetime knee injury had lower previous-week KOOS values than those without an injury history across all five analyzed scores: Symptoms, r = 0.49; seven-item Pain, r = 0.55; Activities of Daily Living, r = 0.51; Sport and Recreation, r = 0.57; and knee-related Quality of Life, r = 0.54 (all *p* < 0.001; Supplementary Material S5). These findings are supportive rather than definitive because KOOS assesses the previous week, whereas the primary outcome represented lifetime injury history. They describe current knee status associated with injury history but do not establish injury timing.

The Pain result was based on the non-standard seven-item score and is not equivalent to the validated nine-item KOOS Pain subscale. Thirty-five participants selected “Not applicable” for one Sport and Recreation item: 30 without and 5 with a reported lifetime knee injury. All completed the remaining four items and retained a calculable score. Full medians, interquartile ranges, effect sizes, *p*-values, and response handling are reported in Supplementary Material S5.

## 4. Discussion

More than half of the analyzed participants reported a knee injury at some point in their lifetime: 56.8% (95% CI 50.8–62.5%). This finding indicates a substantial self-reported history of knee injury within the recruited amateur-sport sample. However, the estimate should not be interpreted as a nationally representative prevalence because recruitment used non-probability convenience sampling, the outcome was based on lifetime self-report, and the study design did not verify diagnoses or establish when the injury occurred relative to current sports participation.

The estimate exceeds the knee injury prevalence reported in previous Saudi studies of male college students in Riyadh and university students in Jazan, where prevalence was 23.2% and 18.0%, respectively [14,15]. Direct comparison should nevertheless be cautious because the present outcome included any self-reported lifetime knee injury, ranging from a bruise to a severe injury, whereas other studies may use narrower, time-limited, or clinically verified definitions. Differences in participant age, sport composition, exposure, and the high proportion of football participants may also contribute.

International studies also show wide variation in knee injury estimates across sports and populations, and a systematic review of adolescent knee injuries similarly reported heterogeneous epidemiological estimates across study settings [9]. This variation is expected because studies differ in sport type, exposure level, injury definition, participant age, competitive level, and whether injuries are clinically verified or self-reported. For example, a small study among East Java fencing athletes identified knee injury in 1 of 14 participants (7.1%) [26], whereas structural knee injuries were reported in 21% of National Basketball Association athletes [27]. Knee injuries represented 47.5% of injuries among Egyptian national handball players [28] and were the most frequent lower-limb injuries among national-squad football players in Sri Lanka [29].

Football was the most robust sport-related association in the study. Its direction and magnitude were broadly consistent in the crude analysis, conventional maximum-likelihood model, full Firth model, bootstrap analysis, and exploratory reduced model. This emphasis on amateur football is also consistent with registry evidence reporting higher ACL rupture risk in amateur than professional football players [30], although ACL rupture is narrower than the broad knee injury outcome used in the present study. The appropriate interpretation in the present study is that current football participation was associated with higher odds of reporting a lifetime knee injury. The cross-sectional design and lifetime outcome prevent causal interpretation; the result may reflect cumulative exposure, continued participation after an earlier injury, or other unmeasured differences between football and non-football participants. The available data did not identify the sport in which each reported injury occurred. Although strengthening exercises were the third most frequently reported activity, they were not clearly associated with self-reported lifetime knee injury history in either the crude or adjusted analysis.

Older age was associated with higher odds of reporting a lifetime knee injury. This may partly reflect a longer cumulative period during which an injury could have occurred, rather than an isolated biological effect of age. Cohort differences, recall patterns, and accumulated sports exposure may also contribute.

The adjusted estimates for male sex and high exercise intensity were above the null, but their confidence intervals were wide and their lower limits were close to 1. These findings should be presented as imprecise exploratory associations rather than established independent risk factors. The male estimate may also be influenced by the small number of female participants, while the high-intensity estimate was based on a relatively small high-intensity category.

The inverse adjusted volleyball coefficient should not be described as a protective effect. Volleyball was not associated with injury in the crude analysis, and 88.4% of volleyball participants also reported football. With overlapping multi-response sport indicators, the adjusted coefficient compares participants who reported volleyball in addition to other modeled sports with otherwise similar participants who did not report volleyball. The estimate may therefore reflect model conditioning, substantial sport overlap, residual confounding, or sampling variability.

Although bruises were the most frequently reported injury type, the healthcare use, pain, and recovery findings indicate that a substantial subset of reported injuries had important clinical and functional consequences. Nearly three-quarters of injured participants reported requiring medical care, approximately half reported physiotherapy, more than one-quarter reported surgical care, and almost half reported recovery for more than four weeks. The post hoc KOOS comparison also showed consistently worse previous-week symptoms and function among participants with a reported lifetime knee injury. This comparison provides supportive cross-sectional evidence of current symptom and functional differences between injury history groups but does not validate the timing, diagnosis, or sport context of the reported injuries.

The present study identified an association between current football participation and lifetime self-reported knee injury history; it did not identify the sport in which the injury occurred or test a prevention intervention. Football-focused neuromuscular warm-up programs, progressive conditioning, injury prevention education, and early rehabilitation access are supported by the external football injury prevention literature rather than by intervention evidence from this study [31]. Together with that external evidence, the current findings may help inform the prioritization and evaluation of prevention and rehabilitation initiatives in amateur football settings. They do not establish that any specific strategy would reduce injuries in this sample.

## 5. Limitations

This study has several important limitations. Its cross-sectional design does not establish temporal sequence or causality; therefore, the findings represent associations rather than causal risk factors. Recruitment used non-probability convenience sampling, and a conventional response rate could not be calculated because the number of individuals who viewed or received the invitation was not recorded. The platform also did not retain the number of individuals who failed the mandatory pre-questionnaire eligibility gateway or the criterion they did not meet. Consequently, the 462 submissions represent only individuals who passed the gateway, and the initial recruitment funnel cannot be quantified completely. Because absence of chronic disease was an eligibility requirement, the findings may not generalize to amateur players with chronic conditions.

The predominance of male participants and football players limits generalizability to female players and less represented sports. Although this imbalance may partly reflect Saudi sport participation patterns, its magnitude limits population-level inference. The final sample did not reach the prespecified recruitment target of 323 after eligibility and consistency exclusions, reducing precision for subgroup, sparse sport, and multivariable estimates. Sparse categories included rare sports, the female reference group, high exercise intensity, daily exercise, and exercise duration greater than 2 h.

Knee injuries were broadly defined, self-reported, and not clinically verified, introducing recall bias, diagnostic heterogeneity, and possible misclassification. The net direction of selection and recall-related bias cannot be determined. Participants with previous knee problems or greater engagement in sport may have been more likely to respond, potentially increasing the observed proportion, whereas remote or minor injuries may have been forgotten. The outcome represented lifetime self-reported history rather than point, annual, or clinically confirmed prevalence. Extreme-case reclassification of the 18 internally inconsistent records changed the estimate by no more than 3.5 percentage points from the primary estimate, but this analysis addressed only one source of uncertainty and did not resolve selection bias or other misclassification. The questionnaire did not record the sport at the time of injury or specify whether follow-up details referred to the most recent, most severe, or another injury.

Sport and training variables described current or usual participation and were not temporally linked to the lifetime injury outcome. Participants could report multiple sports, and primary sport, sport-specific exposure time, footwear, playing surface, warm-up quality, biomechanics, and previous injury history were not fully measured. These limitations affect interpretation of the adjusted sport coefficients and may introduce exposure misclassification. Sport categories were partly derived by harmonizing checkbox and free-text responses. Although direct lexical equivalents and recognizable spelling variants were coded conservatively, some responses could reasonably be classified differently, and residual sport category misclassification remains possible.

The study-specific 26-item questionnaire was developed and administered directly in Arabic, so translation and back-translation were not applicable. It was not formally evaluated through expert-panel content validation, test–retest reliability, or psychometric testing. Although the Saudi Arabic KOOS was previously validated, its validation population consisted of patients with knee osteoarthritis, and its psychometric performance may not transfer fully to younger amateur players with heterogeneous injury histories [17]. Two Pain items, P3 and P7, were omitted during transfer to the online form. The resulting seven-item score is not equivalent to the complete nine-item KOOS Pain subscale and was not compared with published Pain reference values.

One Sport and Recreation item had differently distributed “Not applicable” responses between injury history groups. Although all affected participants completed enough items for scoring, an effect on the Sport and Recreation comparison cannot be excluded. Because KOOS refers to the previous week, the post hoc comparison cannot establish the timing or diagnosis of a lifetime injury. The omitted Pain items may also have affected pain-domain comparability and could overestimate pain-free status.

Multiple crude, adjusted, sensitivity, and post hoc comparisons were conducted without formal multiplicity adjustment. Borderline findings, particularly those for sex, high exercise intensity, volleyball, and cycling, should therefore be considered exploratory and interpreted using effect estimates and confidence intervals rather than the *p*-value threshold alone.

## 6. Conclusions

In this non-probability convenience sample of amateur players in Saudi Arabia, 56.8% reported a lifetime history of knee injury (95% CI 50.8–62.5%). Football participation was the most consistent correlate across the crude, adjusted, and sensitivity analyses. These findings are not causal or nationally representative, and the borderline secondary associations and post hoc KOOS comparison should be considered exploratory. Nevertheless, the self-reported injury characteristics and KOOS comparison indicate that injury history in this sample was associated with substantial current symptom and functional differences. Together with external injury prevention evidence, the findings may help inform the prioritization and evaluation of prevention and rehabilitation initiatives in amateur football settings.

## Figures and Tables

**Table 1:** Demographic characteristics of participants (n = 273).

Variable	Category	n (%)
Age group	18–24 years	44 (16.1%)
	25–34 years	137 (50.2%)
	35–44 years	79 (28.9%)
	45+ years	13 (4.8%)
Sex	Male	244 (89.4%)
	Female	29 (10.6%)
BMI category	Underweight (<18.5 kg/m^2^)	13 (4.8%)
	Normal weight (18.5–24.9 kg/m^2^)	123 (45.1%)
	Overweight (25.0–29.9 kg/m^2^)	82 (30.0%)
	Obese (≥30.0 kg/m^2^)	51 (18.7%)
	Invalid/missing	4 (1.5%)
Region	Central	73 (26.7%)
	North	34 (12.5%)
	South	28 (10.3%)
	East	31 (11.4%)
	West	106 (38.8%)
	Other/unclear	1 (0.4%)

**Table 2:** Sports participation and training characteristics (n = 273).

Variable	Category	n (%)
Sports practiced *	Football	204 (74.7%)
	Running	90 (33.0%)
	Strengthening exercises	86 (31.5%)
	Volleyball	69 (25.3%)
	Cycling	37 (13.6%)
	Walking	20 (7.3%)
	Basketball	15 (5.5%)
	Swimming	11 (4.0%)
	Tennis	7 (2.6%)
	Karate	3 (1.1%)
	Parachuting	2 (0.7%)
	Handball	2 (0.7%)
Weekly exercise frequency	1–3 days	170 (62.3%)
	4–6 days	87 (31.9%)
	Daily	16 (5.9%)
Exercise duration	≤1 h	112 (41.0%)
	1–2 h	141 (51.6%)
	>2 h	20 (7.3%)
Exercise intensity	Low	74 (27.1%)
	Medium	176 (64.5%)
	High	23 (8.4%)
Self-rated health	Very bad	1 (0.4%)
	Weak/poor	7 (2.6%)
	Good	59 (21.6%)
	Very good	116 (42.5%)
	Excellent	90 (33.0%)
Sport participation pattern	Regular sport participation	125 (45.8%)
	Occasional sport participation	148 (54.2%)

* Sports were recorded as overlapping multiple-response indicators; therefore, percentages exceed 100%. Counts incorporate direct lexical equivalents and recognizable spelling variants identified through the conservative coding procedure described in Section 2.4 and Supplementary Material S2.

**Table 3:** Clinical characteristics and consequences of knee injuries among injured participants (n = 155).

Domain	Category	n (%)
Cause of injury	Clash with another player	80 (51.6%)
	No clash	72 (46.5%)
	Not applicable/missing	3 (1.9%)
Type of knee injury *	Bruise	62 (40.0%)
	Meniscus/cartilage	50 (32.3%)
	Anterior cruciate/anterior ligament	45 (29.0%)
	Collateral/side ligament	22 (14.2%)
	Posterior cruciate/posterior ligament	13 (8.4%)
Required medical care	Yes	115 (74.2%)
	No	38 (24.5%)
	Not applicable/missing	2 (1.3%)
Type of medical care	Physiotherapy	77 (49.7%)
	Surgical care	41 (26.5%)
	No medical care needed	36 (23.2%)
	Not applicable/missing	1 (0.6%)
Used crutch for pain relief during movement	Yes	71 (45.8%)
	No	82 (52.9%)
	Not applicable/missing	2 (1.3%)
Pain state	Persistent pain	103 (66.5%)
	Unbearable pain	25 (16.1%)
	No pain	27 (17.4%)
Pain severity score	Mean (SD)	6.5 (2.5)/10
	Median	7/10
	0	3 (1.9%)
	1–3	21 (13.5%)
	4–7	68 (43.9%)
	8–10	63 (40.6%)
Recovery duration	1–3 days	13 (8.4%)
	4–7 days	22 (14.2%)
	1–4 weeks	42 (27.1%)
	>4 weeks	76 (49.0%)
	Not applicable/missing	2 (1.3%)

* Multiple responses were allowed for injury type; therefore, percentages exceed 100%. The type-of-medical-care item was a single-response item reported among injured participants and is shown independently from the preceding yes/no item on whether medical care was required; totals may not align exactly because of item-level missingness and skip-pattern differences.

**Table 4:** Crude associations between participant characteristics and self-reported lifetime knee injury.

Variable	Category	n	Injured, n	Injured, %	cOR	95% CI	Global *p*
Age group	18–24	44	19	43.2	1.00	Reference	0.170
	25–34	137	78	56.9	1.74	0.88–3.45	
	35–44	79	49	62.0	2.15	1.01–4.55	
	45+	13	9	69.2	2.96	0.79–11.09	
Sex	Female	29	9	31.0	1.00	Reference	0.003
	Male	244	146	59.8	3.31	1.45–7.57	
BMI category	<18.5	13	5	38.5	0.37	0.12–1.21	0.097
	18.5–24.9	123	77	62.6	1.00	Reference	
	25.0–29.9	82	40	48.8	0.57	0.32–1.00	
	≥30.0	51	32	62.7	1.01	0.51–1.98	
Exercise frequency	1–3 days/week	170	94	55.3	1.00	Reference	0.132
	4–6 days/week	87	55	63.2	1.39	0.82–2.36	
	Daily	16	6	37.5	0.48	0.17–1.40	
Exercise duration	≤1 h	112	57	50.9	1.00	Reference	0.151
	1–2 h	141	88	62.4	1.60	0.97–2.65	
	>2 h	20	10	50.0	0.96	0.37–2.50	
Exercise intensity	Low	74	38	51.4	1.00	Reference	0.150
	Medium	176	100	56.8	1.25	0.72–2.15	
	High	23	17	73.9	2.68	0.95–7.57	
Sport participation pattern	Occasional sport participation	148	80	54.1	1.00	Reference	0.323
	Regular sport participation	125	75	60.0	1.27	0.79–2.06	

cOR = crude odds ratio; CI = confidence interval. Reference categories are shown as “Reference.” Crude odds ratios and Wald 95% confidence intervals were obtained from univariable logistic-regression models. The reported *p*-value is the overall likelihood-ratio test for each predictor. BMI analyses included 269 participants; all other analyses included 273. Sparse categories, including age 45+, daily exercise, high exercise intensity, the sex comparison with only 9 injured female participants, and exercise duration > 2 h, should be interpreted cautiously.

**Table 5:** Crude associations between commonly reported sports and self-reported lifetime knee injury.

Sport	n	Injured, n	Injured, %	cOR	95% CI	*p*
Football	204	127	62.3	2.42	1.38–4.22	0.002
Running	90	48	53.3	0.81	0.49–1.35	0.421
Strengthening exercises	86	55	64.0	1.54	0.91–2.61	0.103
Volleyball	69	35	50.7	0.72	0.42–1.25	0.242
Cycling	37	25	67.6	1.70	0.81–3.54	0.149

Sport indicators were multi-response and were not mutually exclusive. Odds ratios compare participants reporting each sport with all participants not reporting that sport. Checkbox and free-text responses were harmonized using the conservative coding dictionary described in Section 2.4 and Supplementary Material S2. Crude odds ratios and Wald 95% confidence intervals were obtained from univariable logistic-regression models; *p*-values were obtained using likelihood-ratio tests. Sports were analyzed inferentially only when both outcome cells contained at least 10 participants.

**Table 6:** Sports summarized descriptively because at least one outcome cell contained fewer than 10 participants.

Sport	n	Injured, n	Injured, %
Walking	20	8	40.0
Basketball	15	6	40.0
Swimming	11	6	54.5
Tennis	7	5	71.4
Karate	3	2	66.7
Parachuting	2	1	50.0
Handball	2	2	100.0

No odds ratios or inferential *p*-values are presented for these sparse categories. Counts incorporate direct lexical equivalents and recognizable spelling variants identified in free-text responses according to the conservative coding dictionary described in Section 2.4 and Supplementary Material S2.

**Table 7:** Adjusted associations with self-reported lifetime knee injury: Firth penalized logistic regression (n = 269).

Variable	aOR	95% Profile Penalized-Likelihood CI	*p*
Age (per year)	1.04	1.01–1.08	0.023
Male sex	2.49	1.00–6.47	0.049
BMI (per kg/m^2^)	0.99	0.94–1.05	0.732
4–6 exercise days/week vs. 1–3	1.26	0.66–2.43	0.480
Daily exercise vs. 1–3 days/week	0.72	0.22–2.25	0.566
Exercise duration 1–2 h vs. ≤1 h	1.68	0.92–3.08	0.093
Exercise duration >2 h vs. ≤1 h	1.45	0.47–4.55	0.514
Medium intensity vs. low	0.90	0.45–1.78	0.755
High intensity vs. low	3.53	1.03–13.58	0.045
Regular sport participation vs. Occasional sport participation	0.66	0.34–1.26	0.212
Football	2.60	1.35–5.13	0.004
Running	0.88	0.49–1.60	0.683
Strengthening exercises	1.53	0.83–2.85	0.171
Volleyball	0.54	0.29–1.00	0.049
Cycling	2.05	0.92–4.78	0.080

aOR = adjusted odds ratio; CI = confidence interval. Overall penalized likelihood-ratio test: χ^2^ = 36.29, df = 15, *p* = 0.002. The model included 154 participants with and 115 without the outcome. *p*-values are penalized likelihood-ratio tests. Odds ratios and confidence interval limits are reported to two decimal places; statistical inference was based on unrounded estimates, so a displayed confidence limit may round to 1.00 while the corresponding *p*-value is <0.05. No complete or quasi-complete separation was detected; maximum VIF = 1.74. Sport indicators were overlapping and not mutually exclusive.

## Data Availability

The data presented in this study are available on reasonable request from the corresponding author. The data are not publicly available due to privacy and ethical restrictions.

## Supplementary Materials

The following supporting information can be downloaded at: https://www.mdpi.com/article/10.3390/healthcare14172738/s1, Supplementary Material S1: Study-specific Arabic questionnaire; Supplementary Material S2: Participant eligibility and data cleaning audit; Supplementary Material S3: Maximum-likelihood versus Firth comparison; Supplementary Material S4: Sensitivity analyses; Supplementary Material S5: KOOS values by injury history status. File S1: A completed STROBE checklist. Refs. [17,32,33,34,35,36] are cited in the Supplementary Materials.

## Abbreviations

The following abbreviations are used in this manuscript:

**Abbreviation**	**Definition**
ACL	anterior cruciate ligament
ADL	activities of daily living
aOR	adjusted odds ratio
BMI	body mass index
CI	confidence interval
cOR	crude odds ratio
KOOS	Knee Injury and Osteoarthritis Outcome Score
MUREC	Majmaah University Research Ethics Committee
OR	odds ratio
VIF	variance inflation factor
