# Supplementary material for: The Prevalence and Associated Factors of Self-Reported Knee Injuries Among Amateur Players in Saudi Arabia
Source: Healthcare (Basel). 2026 Aug 27;14(17):2738. doi: 10.3390/healthcare14172738 (PMC13564730; doi:10.3390/healthcare14172738)
Supplement: Supplementary file 1 [file healthcare-14-02738-s001.zip › File S1 STROBE_Checklist_Cross_Sectional_Completed.pdf]

STROBE Statement - Checklist for Cross-Sectional Studies

Manuscript ID: healthcare-4463053

The Prevalence and Associated Factors of Self-Reported Knee Injuries Among Amateur Players in Saudi Arabia

Section	Item	STROBE recommendation	Where reported in the final revised manuscript
Title and abstract	1(a)	Indicate the study design with a commonly used term in the title or abstract.	Abstract: pp. 1–2, lines 24–39 ("cross-sectional").
	1(b)	Provide an informative and balanced abstract summary of what was done and found.	Abstract: pp. 1–2, lines 23–39.
Introduction	2	Explain the scientific background and rationale.	Introduction: pp. 2–3, lines 43–84.
	3	State specific objectives, including prespecified hypotheses.	Objective and statement that no formal directional hypothesis was prespecified: p. 3, lines 74–84.
Methods	4	Present key elements of study design early in the paper.	Section 2.1: p. 3, lines 85–94.
	5	Describe setting, locations, and relevant dates, including recruitment and data-collection periods.	Section 2.1: pp. 3–4, lines 87–119.
	6(a)	Give eligibility criteria and sources and methods of participant selection.	Section 2.1: pp. 3–4, lines 95–119, including the mandatory eligibility gateway, participant-facing information, formal-consent verification, deletion of all questionnaire information from the nine non-consented submissions in accordance with the approved ethics protocol, and retention only of the aggregate refusal count (n = 9); Section 2.2: pp. 4–5, lines 120–154; Figure 1: p. 6 and caption on p. 7, lines 156–159; Supplementary Materials S1–S2. No individual-level or substantive

Section	Item	STROBE recommendation	Where reported in the final revised manuscript
			information from the nine non-consented submissions was included in descriptive, inferential, sensitivity, or supplementary analyses.
	7	Clearly define outcomes, exposures, predictors, potential confounders, and effect modifiers.	Sections 2.3–2.4: pp. 7–11, lines 160–308, including the outcome definition, sport participation pattern, multi-response sport indicators, and harmonization of checkbox and free-text sport responses; Supplementary Material S2, Table S2B.
	8	For each variable, give data sources and assessment methods; describe comparability if more than one group.	Sections 2.3.1–2.3.2: pp. 7–8, lines 161–209; Supplementary Materials S1 and S5.
	9	Describe efforts to address potential sources of bias.	Eligibility, consent, data-cleaning, and consistency procedures: pp. 3–5, lines 95–154; statistical and sensitivity procedures: pp. 8–11, lines 210–308; Limitations: pp. 22–24, lines 545–603.
	10	Explain how study size was arrived at.	Section 2.2: pp. 4–5, lines 120–154, including the pragmatic recruitment target, achieved-sample shortfall, and participant flow.
	11	Explain handling of quantitative variables and chosen groupings.	Section 2.4: pp. 9–10, lines 241–274, including age and BMI handling and conservative classification of free-text sport responses; Tables 1 and 4; Supplementary Material S2, Table S2B.
	12(a)	Describe all statistical methods, including methods	Section 2.4: pp. 8–11, lines 210–308,

Section	Item	STROBE recommendation	Where reported in the final revised manuscript
		used to control for confounding.	including R software and packages, Wilson confidence intervals, univariable logistic regression, Firth penalized logistic regression, diagnostics, bootstrap resampling, KOOS scoring, and bias-reduction procedures; Supplementary Materials S2–S4.
	12(b)	Describe methods used to examine subgroups and interactions.	No subgroup or interaction analyses were prespecified or performed: p. 10, line 256.
	12(c)	Explain how missing data were addressed.	Anthropometric complete-case analysis and no imputation: p. 10, lines 270–274; KOOS available-item handling: pp. 10–11, lines 286–301; Supplementary Material S5.
	12(d)	Describe analytical methods taking account of sampling strategy.	No sampling weights or design-based variance corrections were applied: p. 10, lines 257–258.
	12(e)	Describe sensitivity analyses.	Extreme-case outcome classification: p. 9, lines 249–251; model sensitivity and bootstrap analyses: pp. 19–20, lines 437–454; Supplementary Materials S3–S4.
Results	13(a)	Report numbers at each stage of the study.	Section 2.2: pp. 4–5, lines 120–154; Figure 1: p. 6 and caption on p. 7, lines 156–159; Section 3.1: pp. 11–13, lines 310–334.
	13(b)	Give reasons for non-participation or exclusion at each stage.	Sections 2.1–2.2: pp. 3–5, lines 95–154; Figure 1: p. 6. The number and reasons

Section	Item	STROBE recommendation	Where reported in the final revised manuscript
			for non-progression through the mandatory gateway were not retained; reasons for exclusions among submitted records are reported in Section 2.2 and Supplementary Material S2.
	13(c)	Consider use of a flow diagram.	Figure 1: p. 6; caption: p. 7, lines 156–159; standalone high-resolution figure supplied separately.
	14(a)	Give participant characteristics and information on exposures and potential confounders.	Section 3.1 and Tables 1–2: pp. 11–13, lines 310–334; clinical characteristics in Section 3.2 and Table 3: pp. 13–15, lines 335–361.
	14(b)	Indicate number of participants with missing data for each variable of interest.	BMI missing or implausible data: p. 5, lines 148–154, and Section 3.1, p. 11, lines 310–317; injury-item missing or not-applicable responses: Table 3; KOOS unavailable-item responses: Section 3.7, p. 20, lines 455–469, and Supplementary Material S5.
	15	Report numbers of outcome events or summary measures.	Section 3.2 and Table 3: pp. 13–15, lines 335–361.
	16(a)	Give unadjusted and adjusted estimates and precision; make clear what was adjusted for and why.	Sections 3.3–3.5 and Tables 4–6: pp. 15–19, lines 362–436; predictor rationale and variable handling: p. 10, lines 259–274.
	16(b)	Report category boundaries when continuous variables were categorized.	Tables 1 and 4; Section 2.4: p. 10, lines

Section	Item	STROBE recommendation	Where reported in the final revised manuscript
			266–269.
	16(c)	If relevant, translate relative-risk estimates into absolute risk for a meaningful time period.	Not applicable: the cross-sectional outcome was lifetime self-reported injury history and adjusted estimates were odds ratios without a defined prospective time period.
	17	Report other analyses, including subgroup, interaction, and sensitivity analyses.	Extreme-case prevalence bounds: p. 13, lines 335–343; model stability and post hoc KOOS analyses: pp. 19–20, lines 437–469; Supplementary Materials S3–S5.
Discussion	18	Summarize key results with reference to study objectives.	Discussion: pp. 20–22, lines 470–544.
	19	Discuss limitations, including direction and magnitude of potential bias or imprecision.	Limitations: pp. 22–24, lines 545–603, including the unquantified eligibility gateway, incomplete initial recruitment funnel, restricted generalizability, measurement limitations, and direction and magnitude of potential biases.
	20	Give a cautious overall interpretation considering objectives, limitations, multiplicity, similar studies, and other evidence.	Discussion and Limitations: pp. 20–24, lines 470–603; Conclusions: p. 24, lines 604–614.
	21	Discuss generalisability (external validity).	Discussion and Limitations: pp. 20–24, lines 470–603; Conclusions: p. 24, lines 604–614, including restricted generalizability to participants who passed the eligibility gateway and did not report chronic disease.

Section	Item	STROBE recommendation	Where reported in the final revised manuscript
Other information	22	Give the source of funding and the role of the funders.	Funding statement: p. 25, line 625 (no external funding).

Note: Page and line references correspond to the final revised manuscript supplied with these submission files.
