# Supplementary material for: The Prevalence and Associated Factors of Self-Reported Knee Injuries Among Amateur Players in Saudi Arabia
Source: Healthcare (Basel). 2026 Aug 27;14(17):2738. doi: 10.3390/healthcare14172738 (PMC13564730; doi:10.3390/healthcare14172738)
Supplement: Supplementary file 1 [file healthcare-14-02738-s001.zip › Supplementary_Material_S1_Questionnaire.pdf]

Knee Injury Questionnaire among Amateur Athletes in the Kingdom of Saudi Arabia — English Translation (Original Arabic version used in the study: see page 6)

Study Questionnaire — Researcher-Specific Questions

Participant-facing introductory text displayed electronically before the questionnaire items: “This questionnaire was designed to learn your views about your knee. The information will help us understand how you feel about your knee and how able you are to perform your usual activities. Answer each question by marking the appropriate box, and select only one box for each question unless otherwise indicated. If you are unsure of an answer, select the closest appropriate response.”

Procedural description of the eligibility and consent gate (not part of the 26-item questionnaire): Before opening the questionnaire, the participant was required to confirm that all of the following eligibility criteria applied: (1) age 18 to 60 years, (2) amateur athlete, (3) current participation in a sport or physical activity, (4) no chronic disease, and (5) willingness to provide informed consent. The questionnaire opened only for those who confirmed that all criteria applied; those who did not confirm them could not proceed. The platform did not retain the number of people who did not proceed or which criterion did not apply to them. A “No” response to formal consent item 1 did not technically terminate the form; it was treated as refusal of consent. Because the form remained open, fields entered after item 1 initially appeared in the raw export. All questionnaire information from the nine non-consented submissions was subsequently deleted, consistent with the approved ethics protocol. No individual-level or substantive information from these submissions was included in descriptive, inferential, sensitivity, or supplementary analyses. Only the aggregate number of consent refusals (n = 9) was retained for participant-flow and exclusion reporting.

Section 1: Consent and Basic Information

1. I confirm my consent to participate in the research					
☐ Yes	☐ No				

2. Age (years)					
....					

3. Sex					
☐ Male	☐ Female				

4. Weight (kg)					
....					

5. Height (cm)					
....					

6. Region					
....					

7. What is your sports level?					
☐ Amateur	☐ Professional	☐ I do not participate in sports			

8. What types of sports do you participate in? (More than one answer may be selected)					
☐ Football	☐ Volleyball	☐ Basketball	☐ Handball	☐ Tennis	☐ Strengthening exercises
☐ Cycling	☐ Running	☐ Walking	☐ Swimming	☐ Karate	☐ Skydiving
☐ Other (specify):					

9. How many days do you participate in sports?					
☐ 1 to 3 days	☐ 4 to 6 days	☐ 7 days per week			

10. How long do you spend participating in sports?					
☐ One hour or less	☐ One to two hours	☐ Two hours or more			

11. Exercise intensity level?					
☐ Low	☐ Moderate	☐ High			

Section 2: Health Status, Injury History, Treatment, and Prevention

12. How do you rate your health status?					
☐ Excellent	☐ Very good	☐ Good	☐ Poor	☐ Bad	

**13. What is your physical activity pattern? [Term used in the analysis and report: Sport participation pattern]**					
☐ Professional athlete	☐ Regular sport participation	☐ Occasional sport participation	☐ I do not participate in sports		

**14. Have you ever sustained a knee injury? (Any knee injury, from a bruise to the most severe injuries)**					
☐ Yes	☐ No				

**15. Was the injury caused by contact with another player during sports activity?**					
☐ Yes	☐ No	☐ Not applicable (I did not sustain an injury)			

**16. What type of knee injury did you sustain? (More than one answer may be selected)**					
☐ Bruise	☐ Meniscus	☐ Anterior ligament	☐ Posterior ligament	☐ Collateral ligament	☐ Not applicable (I did not sustain an injury)
☐ Other (specify):					

**17. Did your injury require health care?**					
☐ Yes	☐ No	☐ Not applicable (I did not sustain an injury)			

**18. What type of medical care?**					
☐ Physiotherapy	☐ Surgical intervention	☐ I did not need medical care	☐ Not applicable (I did not sustain an injury)		

**19. Did you use crutches to reduce knee pain while moving?**					
☐ Yes	☐ No	☐ Not applicable (I did not sustain an injury)			

**20. Rate your pain severity, where 0 means no pain and 10 means unbearable pain**					
☐ 0	☐ 1	☐ 2	☐ 3	☐ 4	☐ 5
☐ 6	☐ 7	☐ 8	☐ 9	☐ 10	

**21. Pain status**					
☐ No pain	☐ Continuous pain	☐ Unbearable pain			

22. How long did it take to recover from the pain?					
☐ 1 to 3 days	☐ 4 to 7 days	☐ 1 to 4 weeks	☐ More than 4 weeks	☐ Not applicable (I did not sustain an injury)	

23. Is the pain associated with a specific movement? (Please specify)					
....					

24. How do you protect yourself from injury? (More than one answer may be selected)					
☐ Warm-up exercises	☐ Stretching	☐ Use of appropriate equipment	☐ Awareness of body movement	☐ Training	

25. Did you treat the injury using any of the following methods? (More than one answer may be selected)					
☐ Applying ice	☐ Elevating the injured leg	☐ Using analgesics	☐ Using a knee brace	☐ Not applicable (I did not sustain an injury)	

26. Can you use the full range of motion of the knee?					
☐ Yes	☐ No				

In addition to the above, the participant completes the Arabic KOOS — Knee Injury and Osteoarthritis Outcome Score.
The researchers obtained permission to use the instrument from Mapi Research Trust — the authorized licensor and distributor of the instrument on behalf of the copyright owner — under User License Agreement No. 142688.

KOOS© Ewa Maria Roos, 1998
KOOS contact information and permission to use: Mapi Research Trust, Lyon, France, https://eprovide.mapi-trust.org

References

Reference citations for the instrument in accordance with the license agreement:

Roos EM, Roos HP, Lohmander LS, Ekdahl C, Beynnon BD. Knee Injury and Osteoarthritis Outcome Score (KOOS) — development of a self-administered outcome measure. J Orthop Sports Phys Ther. 1998;28:88–96.

Roos EM, Roos HP, Ekdahl C, Lohmander LS. Knee injury and Osteoarthritis Outcome Score (KOOS) — validation of a Swedish version. Scand J Med Sci Sports. 1998;8:439–448.

Roos EM, Toksvig-Larsen S. Knee injury and Osteoarthritis Outcome Score (KOOS) — validation and comparison to the WOMAC in total knee replacement. Health Qual Life Outcomes. 2003;1:17.

Macri EM, Young JJ, Ingelsrud LH, Khan KM, Terluin B, Juhl CB, Whittaker JL, Culvenor AG, Crossley KM, Roos EM. Meaningful thresholds for patient-reported outcomes following interventions for anterior cruciate ligament tear or traumatic meniscus injury: a systematic review for the OPTIKNEE consensus. Br J Sports Med. 2022;56(24):1432–1444.

Roos EM. 30 years with the Knee injury and Osteoarthritis Outcome Score (KOOS). Osteoarthritis Cartilage. 2024;32(4):421–429. doi:10.1016/j.joca.2023.10.002.

Additional Saudi Arabic version used:

Alfadhel SA, Vennu V, Alnahdi AH, Omar MT, Alasmari SH, AlJafri Z, Bindawas SM. Cross-cultural adaptation and validation of the Saudi Arabic version of the Knee Injury and Osteoarthritis Outcome Score (KOOS). Rheumatol Int. 2018;38:1547–1555. https://doi.org/10.1007/s00296-018-4072-7

Thank you very much for answering all the questions in this questionnaire.

استبانة إصابات الركبة لدى اللاعبين الهواة في المملكة العربية السعودية

استبانة الدراسة — الأسئلة الخاصة بالباحثين

النص التعريفي الذي ظهر للمشاركين إلكترونياً قبل أسئلة الاستبانة: «هذه الاستبانة صُممت لمعرفة رأيك حول ركبتك. المعلومات سوف تساعدنا في معرفة شعورك بركبتك وكيف ستكون قادراً على أداء نشاطاتك الاعتيادية. أجب عن كل سؤال بوضع علامة في المربع المناسب، واختر مربعاً واحداً فقط لكل سؤال ما لم يُذكر خلاف ذلك. إذا كنت غير متأكد من الإجابة اختر أقرب إجابة مناسبة».

وصف إجرائي لبوابة الأهلية والموافقة (ليست جزءاً من الاستبانة ذات 26 بنداً): قبل فتح الاستبانة، كان على المشارك تأكيد انطباق جميع معايير الأهلية الآتية عليه: (1) العمر من 18 إلى 60 سنة، (2) أن يكون لاعباً هاوياً، (3) المشاركة الحالية في رياضة أو نشاط بدني، (4) عدم وجود مرض مزمن، و(5) الاستعداد لتقديم الموافقة المستنيرة. لم تُفتح الاستبانة إلا لمن أكد انطباق جميع المعايير، ولم يتمكن من لم يؤكدها من المتابعة. لم تحتفظ المنصة بعدد الأشخاص الذين لم يتابعوا أو بالمعيار الذي لم ينطبق عليهم. سُجلت الموافقة المستنيرة رسمياً مرة أخرى في البند الأول من الاستبانة. لم تؤدِّ إجابة «لا» في البند الأول إلى إنهاء النموذج تقنياً، لكنها عُدّت رفضاً للموافقة. ولأن النموذج بقي مفتوحاً، ظهرت أي حقول أُدخلت بعد البند الأول مبدئياً في ملف التصدير الخام. حُذفت لاحقاً جميع معلومات الاستبانة الخاصة بالتسعة الذين لم يقدموا الموافقة، بما يتفق مع البروتوكول الأخلاقي المعتمد، ولم تُدرج أي معلومات فردية أو موضوعية منهم في أي تحليلات وصفية أو استدلالية أو تحليلات حساسية أو مواد تكميلية. اقتصر الاحتفاظ على العدد الإجمالي لحالات رفض الموافقة (ن = 9) لأغراض مخطط تدفق المشاركين وتقرير الاستبعادات.

القسم الأول: الموافقة والبيانات الأساسية

**1. أقر بالموافقة على المشاركة في البحث**					
☐ نعم	☐ لا				

**2. العمر (بالسنوات)**					
....					

**3. الجنس**					
☐ ذكر	☐ أنثى				

**4. الوزن (كجم)**					
....					

**5. الطول (سم)**					
....					

**6. المنطقة**					
....					

**7. ما هو مستواك الرياضي؟**					
☐ هاوي	☐ محترف	☐ لا أمارس الرياضة			

8. أنواع الرياضة التي تمارسها؟ (يمكن اختيار أكثر من إجابة)					
☐ كرة القدم	☐ كرة الطائرة	☐ كرة السلة	☐ كرة اليد	☐ كرة التنس	☐ تمارين التقوية
☐ دراجة هوائية	☐ الجري	☐ المشي	☐ السباحة	☐ الكاراتيه	☐ قفز مظلي
☐ أخرى (اذكرها):					

9. كم عدد الأيام التي تمارس فيها الرياضة؟					
☐ يوم إلى 3 أيام	☐ 4 إلى 6 أيام	☐ 7 أيام بالأسبوع			

10. كم المدة التي تقضيها في ممارسة الرياضة؟					
☐ ساعة أو أقل	☐ ساعة إلى ساعتين	☐ ساعتان أو أكثر			

11. مستوى شدة التمرين؟					
☐ خفيف	☐ متوسط	☐ عالي			

القسم الثاني: الحالة الصحية وتاريخ الإصابة والعلاج والوقاية

12. كيف تقيّم حالتك الصحية؟					
☐ ممتازة	☐ جيدة جداً	☐ جيدة	☐ ضعيفة	☐ سيئة	

13. معدل نشاطك البدني؟ [المسمى المستخدم في التحليل والتقرير: Sport participation pattern]					
☐ رياضي محترف	☐ شخص رياضي [Regular sport participation]	☐ أمارس الرياضة أحياناً [Occasional sport participation]	☐ لا أمارس الرياضة		

14. هل سبق وتعرضت لإصابة في الركبة؟ (أي إصابة تعرضت لها الركبة ابتداءً من الكدمة إلى أشد الإصابات)					
☐ نعم	☐ لا				

15. هل الإصابة بسبب اشتباك مع لاعب آخر أثناء النشاط الرياضي؟					
☐ نعم	☐ لا	☐ لا ينطبق (لم أتعرض لإصابة)			

16. نوع الإصابة التي تعرضت لها بالركبة؟ (يمكن اختيار أكثر من إجابة)					
☐ كدمة	☐ غضروف هلالي	☐ رباط أمامي	☐ رباط خلفي	☐ رباط جانبي	☐ لا ينطبق (لم أتعرض لإصابة)

**16. نوع الإصابة التي تعرضت لها بالركبة؟ (يمكن اختيار أكثر من إجابة)**					
☐ أخرى (اذكرها):					

**17. هل كانت إصابتك تحتاج إلى رعاية صحية؟**					
☐ نعم	☐ لا	☐ لا ينطبق (لم أتعرض لإصابة)			

**18. ما نوع الرعاية الطبية؟**					
☐ علاج طبيعي	☐ تدخل جراحي	☐ لم أكن بحاجة لرعاية طبية	☐ لا ينطبق (لم أتعرض لإصابة)		

**19. هل استخدمت عكازاً لتخفيف ألم الركبة أثناء الحركة؟**					
☐ نعم	☐ لا	☐ لا ينطبق (لم أتعرض لإصابة)			

**20. تقديرك لشدة الألم، حيث إن صفر يعني عدم وجود ألم بينما 10 ألم لا يطاق**					
☐ 0	☐ 1	☐ 2	☐ 3	☐ 4	☐ 5
☐ 6	☐ 7	☐ 8	☐ 9	☐ 10	

**21. حالة الألم**					
☐ لا يوجد ألم	☐ ألم مستمر	☐ ألم لا يطاق			

**22. كم كانت مدة التعافي من الألم؟**					
☐ يوم إلى 3 أيام	☐ 4 إلى 7 أيام	☐ أسبوع إلى 4 أسابيع	☐ أكثر من 4 أسابيع	☐ لا ينطبق (لم أتعرض لإصابة)	

**23. هل الألم مرتبط بحركة محددة؟ (اذكرها)**					
....					

**24. كيف تقي نفسك من الإصابة؟ (يمكن اختيار أكثر من إجابة)**					
☐ تمارين الإحماء	☐ الإطالة	☐ استخدام الأجهزة المناسبة	☐ الشعور بحركة الجسم	☐ التدريب	

**25. هل قمت بعلاج الإصابة بأحد الطرق التالية؟ (يمكن اختيار أكثر من إجابة)**					
☐ وضع الثلج	☐ جعل القدم المصابة مرتفعة	☐ استخدام المسكنات	☐ استخدام رباط الركبة	☐ لا ينطبق (لم أتعرض لإصابة)	

**26. هل تستطيع استخدام المدى الحركي لكامل الركبة؟**					
☐ نعم	☐ لا				

إضافة إلى ما سبق، يعبّئ المشارك نموذج KOOS العربي — مقياس إصابات الركبة والالتهاب المفصلي العظمي (Knee Injury and Osteoarthritis Outcome Score).

وقد حصل الباحثون على ترخيص استخدام المقياس من Mapi Research Trust — الجهة المرخِّصة والموزِّعة للمقياس نيابةً عن مالكة حقوقه — بموجب اتفاقية ترخيص المستخدم رقم 142688.

KOOS© Ewa Maria Roos, 1998

KOOS contact information and permission to use: Mapi Research Trust, Lyon, France, https://eprovide.mapi-trust.org

المراجع

المراجع المرجعية للمقياس وفق اتفاقية الترخيص:

Roos EM, Roos HP, Lohmander LS, Ekdahl C, Beynnon BD. Knee Injury and Osteoarthritis Outcome Score (KOOS) — development of a self-administered outcome measure. J Orthop Sports Phys Ther. 1998;28:88–96.

Roos EM, Roos HP, Ekdahl C, Lohmander LS. Knee injury and Osteoarthritis Outcome Score (KOOS) — validation of a Swedish version. Scand J Med Sci Sports. 1998;8:439–448.

Roos EM, Toksvig-Larsen S. Knee injury and Osteoarthritis Outcome Score (KOOS) — validation and comparison to the WOMAC in total knee replacement. Health Qual Life Outcomes. 2003;1:17.

Macri EM, Young JJ, Ingelsrud LH, Khan KM, Terluin B, Juhl CB, Whittaker JL, Culvenor AG, Crossley KM, Roos EM. Meaningful thresholds for patient-reported outcomes following interventions for anterior cruciate ligament tear or traumatic meniscus injury: a systematic review for the OPTIKNEE consensus. Br J Sports Med. 2022;56(24):1432–1444.

Roos EM. 30 years with the Knee injury and Osteoarthritis Outcome Score (KOOS). Osteoarthritis Cartilage. 2024;32(4):421–429. doi:10.1016/j.joca.2023.10.002.

النسخة العربية السعودية المستخدمة الإضافية:

Alfadhel SA, Vennu V, Alnahdi AH, Omar MT, Alasmari SH, AlJafri Z, Bindawas SM. Cross-cultural adaptation and validation of the Saudi Arabic version of the Knee Injury and Osteoarthritis Outcome Score (KOOS). Rheumatol Int. 2018;38:1547–1555. https://doi.org/10.1007/s00296-018-4072-7

شكراً جزيلاً لإجابتك على كل الأسئلة في هذه الاستبانة.
