# Supplementary material for: The Prevalence and Associated Factors of Self-Reported Knee Injuries Among Amateur Players in Saudi Arabia
Source: Healthcare (Basel). 2026 Aug 27;14(17):2738. doi: 10.3390/healthcare14172738 (PMC13564730; doi:10.3390/healthcare14172738)
Supplement: Supplementary file 1 [file healthcare-14-02738-s001.zip › Supplementary_Material_S2_Eligibility_Data_Cleaning_Audit.pdf]

Supplementary Table S2. Participant eligibility, data-cleaning, and sport-coding audit.

Mandatory pre-questionnaire eligibility gateway	Not captured
Questionnaire submissions received after eligibility gateway	462
Did not provide formal informed consent (questionnaire information deleted; aggregate refusal count retained only)	9
Did not meet amateur-status criterion	129
– reported professional sporting level	66
– reported not practicing sport	63
Sport participation pattern inconsistent with amateur participation	20
– reported professional-athlete status	11
– reported not practicing sport	9
Age outside 18–60 years or invalid age entry	13
**Eligible records**	**291**
Excluded – internally inconsistent injury response	18
- denied knee injury but reported a clash mechanism	8
- denied knee injury but supplied other substantive injury information	10
**Primary analytic sample**	**273**
Excluded from adjusted model – missing/implausible BMI	4
**Adjusted-model sample (Firth)**	**269**

Before questionnaire access, prospective participants were required to affirm a single mandatory eligibility statement covering age 18–60 years, amateur player status, current participation in sport or physical activity, absence of chronic disease, and willingness to provide informed consent. Only individuals who affirmed all criteria could open the questionnaire. The platform did not retain the number who did not proceed or the specific criterion they did not meet. Accordingly, this audit begins with the 462 questionnaire submissions received after the gateway. The online form displayed the exact Arabic participant-information text reproduced in Supplementary Material S1. Formal consent, age, amateur status, current sport participation, and sport participation pattern were subsequently verified using variables available within the submitted-record dataset. A "No" response to formal consent item 1 did not technically terminate the form; it was treated as refusal of consent. Because the form remained open, fields entered after item 1 initially appeared in the raw export. All questionnaire information from the nine non-consented submissions was subsequently deleted, consistent with the approved ethics protocol. No individual-level or substantive information from these submissions was included in descriptive, inferential, sensitivity, or supplementary analyses. Only the aggregate number of consent refusals (n = 9) was retained for participant-flow and exclusion reporting. Within the submitted-record dataset, exclusions were applied hierarchically in the order shown; each record was counted once under the first applicable criterion. All 18 internally inconsistent records answered "No" to the screening question, "Have you ever had a knee injury?", but supplied contradictory injury-related information in subsequent items. Eight recorded that an injury had resulted from a clash with another player. The remaining ten supplied other substantive injury-related information, including a named injury type, healthcare or physiotherapy, persistent pain, recovery details, or treatment information. Across both patterns, several free-text descriptions referred to non-knee or non-specific injuries, including hand or finger, shoulder, and adductor injuries. In all 18 records, the primary knee-injury outcome could not be classified reliably, so the records were excluded from the primary analysis. Four records were excluded from the adjusted model because anthropometric data were unusable: two contained non-numeric text in the height field, one recorded a height of 70 cm, and one recorded a weight of 780 kg. Two further participants entered height in meters ("1.84" and "1,73m"); these values were rescaled to centimeters and retained.

Free-text sport harmonization. The combined sport-response field was searched using exact fixed-string matching. A participant was assigned to a listed sport when the response contained the checkbox label or one of the exact matching terms shown in Supplementary Table S2B. Mixed responses retained every applicable sport category. Direct lexical equivalents and recognizable spelling variants were assigned to the corresponding listed sport, whereas distinct modalities were retained as other activities. The final descriptive counts were 86 for strengthening exercises, 20 for walking, 11 for swimming, and 2 for handball.

Supplementary Table S2B. Final coding dictionary for harmonized free-text sport responses.

Final category	Exact matching terms	Coding decision and raw-response examples	Final n
Strengthening exercises	(Strengthening exercises) تمارين التقوية<br>رفع الاثقال (Lifting Weights)<br>(Iron) حديد<br>(Resistance) المقاومة<br>(Bodybuilders) كمال أجسام<br>(Muscle Building) بناءً العضلات<br>(Bodybuilders) كمال اجسام<br>(Cargo) اثقال<br>(Resistance Exercises)تمارين مقاومه<br>(Lifting Weights) رفع أثقال<br>(Iron)الحديد<br>Bodybuilding	Assigned when any listed exact string occurred. Mixed responses retained all applicable sport categories.	86
Walking	(Walking) المشي<br>(Walk) مشي<br>(Walking) المشيء	Examples assigned include "مشي" and "مشي وكرة خفيفة". ("walk" and "walk and light ball".)	20
Swimming	(Swimming)السباحة<br>(Swimming)السباحه	Examples assigned include "الغوص و السباحة" and "دراجة هوائية;الجري;السباحه، اجهزة الكارديو". ("Diving and Swimming" and "Aerobic; Running; Swimming, Cardio Devices".)	11
Handball	(Handball) كرة اليد<br>(Handball)كرة يد	Examples assigned include "كرة القدم;وكرة اليد تأسيس نادي النجوم" and "كرة يد". (Football; Handball Establishment of the Star Club" and "Handball".)	2
Retained as other activities	كروس فت(CrossFit)<br>(CrossFit) كروسفت<br>كاليستثينكس (Calisthenics)	These were treated as distinct exercise modalities and were not reassigned to strengthening exercises.	3

Matching was applied to the final n = 273 analytic dataset using case-sensitive fixed-string searches. The term-level specification is also reproduced in the accompanying R script. The "Retained as other activities" count reports the three records containing those distinct modalities; it is not an additional mutually exclusive sport category in the main analysis.
