# Supplementary material for: The Prevalence and Associated Factors of Self-Reported Knee Injuries Among Amateur Players in Saudi Arabia
Source: Healthcare (Basel). 2026 Aug 27;14(17):2738. doi: 10.3390/healthcare14172738 (PMC13564730; doi:10.3390/healthcare14172738)
Supplement: Supplementary file 1 [file healthcare-14-02738-s001.zip › Supplementary_Material_S3_ML_vs_Firth.pdf]

Supplementary Table S3. Comparison of maximum-likelihood and Firth penalized logistic regression (n = 269).

Variable	ML aOR	ML 95% CI	ML *p*	Firth aOR	Firth 95% CI	Firth *p*
Age (per year)	1.05	1.01–1.09	0.021	1.04	1.01–1.08	0.023
Male sex	2.71	1.03–7.16	0.044	2.49	1.00–6.47	0.049
BMI (per kg/m²)	0.99	0.94–1.05	0.737	0.99	0.94–1.05	0.732
4–6 exercise days/week vs 1–3	1.28	0.66–2.51	0.465	1.26	0.66–2.43	0.480
Daily exercise vs 1–3 days/week	0.69	0.21–2.31	0.546	0.72	0.22–2.25	0.566
Exercise duration 1–2 h vs ≤1 h	1.73	0.93–3.23	0.085	1.68	0.92–3.08	0.093
Exercise duration >2 h vs ≤1 h	1.49	0.46–4.84	0.504	1.45	0.47–4.55	0.514
Medium intensity vs low	0.88	0.43–1.80	0.734	0.90	0.45–1.78	0.755
High intensity vs low	4.04	1.05–15.50	0.042	3.53	1.03–13.58	0.045
Regular sport participation vs Occasional sport participation	0.64	0.33–1.26	0.199	0.66	0.34–1.26	0.212
**Football**	2.78	1.39–5.56	0.004	2.60	1.35–5.13	0.004
Running	0.88	0.48–1.61	0.681	0.88	0.49–1.60	0.683
Strengthening exercises	1.58	0.84–2.99	0.158	1.53	0.83–2.85	0.171
Volleyball	0.52	0.28–0.99	0.045	0.54	0.29–1.00	0.049
Cycling	2.19	0.93–5.14	0.073	2.05	0.92–4.78	0.080

Adjusted odds ratios (aOR) with 95% confidence intervals (CI) for associations with self-reported lifetime knee injury after conservative harmonization of direct free-text sport equivalents. Maximum-likelihood estimates use Wald confidence intervals and Wald *p*-values; Firth estimates use profile penalized-likelihood confidence intervals and penalized likelihood-ratio *p*-values. Statistical inference used unrounded estimates; displayed confidence limits may round to 1.00. The Firth estimates showed modest shrinkage toward the null, with no reversal in coefficient direction. Sport indicators are multi-response and not mutually exclusive.
