# Supplementary material for: The Prevalence and Associated Factors of Self-Reported Knee Injuries Among Amateur Players in Saudi Arabia
Source: Healthcare (Basel). 2026 Aug 27;14(17):2738. doi: 10.3390/healthcare14172738 (PMC13564730; doi:10.3390/healthcare14172738)
Supplement: Supplementary file 1 [file healthcare-14-02738-s001.zip › Supplementary_Material_S4_Sensitivity_Analyses.pdf]

Supplementary Table S4. Sensitivity analyses: reduced model and bootstrap stability.

Panel A. Reduced Firth penalized logistic regression model (n = 269).

Variable	aOR	95% CI	*p*
Age (per year)	1.04	1.00–1.07	0.038
Male sex	2.42	1.01–6.08	0.046
BMI (per unit)	1.01	0.96–1.06	0.800
High intensity vs low/medium	3.84	1.41–12.44	0.008
**Football**	2.20	1.20–4.11	0.011

Overall penalized likelihood-ratio test: $\chi^2 = 24.19$, df = 5, $p < 0.001$. The intensity contrast in this reduced model is high versus low and medium combined, which differs from the full model (high versus low); the two intensity estimates are therefore not directly comparable. Smaller-outcome observations per estimated parameter = 23.0. Statistical inference was based on unrounded estimates; confidence-interval limits are displayed to two decimal places and may therefore round to 1.00 while the corresponding *p*-value is <0.05.

Panel B. Bootstrap stability of key coefficients (1000 resamples, Firth model refitted in each replicate).

Variable	Median OR	2.5–97.5% interval	% resamples OR > 1
Age (per year)	1.05	1.00–1.09	98.4
Male sex	2.66	1.01–8.18	97.5
High intensity vs low	3.87	1.10–19.89	98.0
**Football**	2.84	1.43–5.98	99.9
Volleyball	0.53	0.28–0.97	2.0

Odds ratios were obtained by refitting the full Firth penalized model within each of 1000 bootstrap resamples after conservative harmonization of direct free-text sport equivalents (random seed 20260724). Football exceeded the null in 999 of 1000 resamples. The high-intensity estimate was directionally stable but imprecise, spanning a broad interval. The inverse volleyball estimate may reflect conditioning arising from overlapping multi-response sport indicators, residual confounding, or sampling variability. Given that 61 of 69 volleyball participants also reported football, it should not be interpreted as evidence of a protective effect. Statistical inference used unrounded estimates; displayed confidence limits may round to 1.00.
