# Supplementary material for: The Prevalence and Associated Factors of Self-Reported Knee Injuries Among Amateur Players in Saudi Arabia
Source: Healthcare (Basel). 2026 Aug 27;14(17):2738. doi: 10.3390/healthcare14172738 (PMC13564730; doi:10.3390/healthcare14172738)
Supplement: Supplementary file 1 [file healthcare-14-02738-s001.zip › Supplementary_Material_S5_KOOS.pdf]

**Supplementary Table S5. Knee Injury and Osteoarthritis Outcome Score (KOOS) scores by self-reported lifetime knee-injury status.**

| KOOS subscale | No reported injury (n = 118) | Reported injury (n = 155) | r | *p* |
|---|---|---|---|---|
| Symptoms (7 items) | 92.9 (85.7–99.1) | 78.6 (64.3–89.3) | 0.49 | <0.001 |
| Pain score (7 administered items) | 100.0 (92.9–100.0) | 82.1 (64.3–92.9) | 0.55 | <0.001 |
| Activities of Daily Living (17 items) | 100.0 (97.1–100.0) | 89.7 (74.3–95.6) | 0.51 | <0.001 |
| Sport and Recreation (5 items) | 100.0 (90.0–100.0) | 70.0 (45.0–90.0) | 0.57 | <0.001 |
| Knee-related Quality of Life (4 items) | 93.8 (81.2–100.0) | 56.2 (37.5–81.2) | 0.54 | <0.001 |

Values are median (interquartile range) on the 0–100 KOOS metric, where 100 indicates no knee problems. Groups were compared using the Mann–Whitney U test; effect size $r = |Z|/\sqrt{N}$. Two of the nine standard Pain items were inadvertently omitted when the paper questionnaire was transferred to the online form. The Pain score was therefore calculated from the seven administered items. It is a non-standard partial score, is not equivalent to the validated nine-item KOOS Pain subscale, and was not compared with published Pain reference values. The four fully administered KOOS subscales and the seven-item Pain score met the minimum available-item requirement used for scoring; no participant-level score was excluded because of insufficient item responses. One Sport and Recreation item concerning twisting or pivoting on the affected knee received a "Not applicable" response from 35 participants: 30 without and 5 with a reported lifetime knee injury. These responses were treated as unavailable item responses; all affected participants completed the remaining four items and therefore satisfied the scoring requirement. KOOS items refer to the previous week and describe current knee status rather than the circumstances of any past injury.
